# Partnership-defined quality approach to companionship during labour and birth in East New Britain, Papua New Guinea: A mixed-methods study

**DOI:** 10.1371/journal.pgph.0000102

**Published:** 2022-02-28

**Authors:** Alyce N. Wilson, Pele Melepia, Rose Suruka, Priscah Hezeri, Dukduk Kabiu, Delly Babona, Pinip Wapi, Meghan A. Bohren, Joshua P. Vogel, Angela Kelly-Hanku, Alison Morgan, James G. Beeson, Christopher Morgan, Naomi Spotswood, Michelle J. L. Scoullar, Lisa M. Vallely, Caroline S. E. Homer

**Affiliations:** 1 Maternal, Child and Adolescent Health Program, Burnet Institute, Melbourne, Australia; 2 Nossal Institute for Global Health, School of Population and Global Health, University of Melbourne, Melbourne, Australia; 3 Healthy Mothers, Healthy Babies, Burnet Institute, Kokopo, Papua New Guinea; 4 St Mary’s Hospital, Kokopo, Papua New Guinea; 5 Nonga General Hospital, Rabaul, Papua New Guinea; 6 Gender and Women’s Health Unit, Centre for Health Equity, School of Population and Global Health, University of Melbourne, Melbourne, Australia; 7 Papua New Guinea Institute for Medical Research, Goroka, Papua New Guinea; 8 Kirby Institute, University of New South Wales, New South Wales, Australia; 9 Global Financing Facility, World Bank, Washington, DC, United States of America; 10 Department of Medicine, University of Melbourne, Melbourne, Australia; 11 Jhpiego, The Johns Hopkins University affiliate, Baltimore, Maryland, United States of America; 12 Department of Paediatrics, Royal Hobart Hospital, Tasmania, Australia; Simon Fraser University, CANADA

## Abstract

Companionship during labour and birth is a critical component of quality maternal and newborn care, resulting in improved care experiences and better birth outcomes. Little is known about the preferences and experiences of companionship in Papua New Guinea (PNG), and how it can be implemented in a culturally appropriate way. The aim of this study was to describe perspectives and experiences of women, their partners and health providers regarding labour and birth companionship, identify enablers and barriers and develop a framework for implementing this intervention in PNG health facilities. A mixed methods study was conducted with five facilities in East New Britain, PNG. Data included 5 facility audits, 30 labour observations and 29 in-depth interviews with women who had recently given birth, partners and maternity care providers. A conceptual framework was developed drawing on existing quality care implementation frameworks. Women and partners wanted companions to be present, whilst health providers had mixed views. Participants described benefits of companionship including encouragement and physical support for women, better communication and advocacy, improved labour outcomes and assistance with workforce issues. Adequate privacy and space constraints were highlighted as key barriers to address. Of the women observed, only 30% of women had a companion present during labour, and 10% had a companion at birth. A conceptual framework was used to highlight the interconnected inputs required at community, facility and provincial health system levels to improve the quality of care. Key elements to address included attitudes towards companionship, the need for education and training and restrictive hospital policies. Supporting women to have their companion of choice present during labour and birth is critical to improving women’s experiences of care and improving the quality of maternal and newborn care. In order to provide companionship during labour and birth in PNG, a complex, intersecting, multi-faceted approach is required.

## Introduction

The presence of a companion (whether a partner, family member, friend or doula) to support a woman during labour and birth is a critical component of good quality maternal and newborn care [1]. Companions help by providing emotional and physical support, assisting women in managing their pain, improving communication with health providers, and advocating for the person giving birth [2]. The WHO Quality of Maternal and Newborn Care Framework highlights labour companions as an important component of quality care [3] and the World Health Organization (WHO) recommends that all women have the opportunity to be supported by a companion [4]. Companionship can be continuous throughout labour and birth or only during labour or after birth [1]. The presence of a companion can both improve the birthing experience, and lead to improved health outcomes for the woman and infant [1,5]. For example, the presence of a companion has been found to reduce the experience of pain, the need for pain relief and facilitates progress in labour. It has been shown to result in less need for intervention during labour, higher breastfeeding rates and potentially lower rates of mistreatment by health care workers [1,6]. Global research is increasingly reaffirming the value of companionship during labour and birth [7–9]. However, in many settings labour companionship is not permitted due to limited health service and health worker support for companionship, inadequate facility infrastructure to maintain privacy, lack of resources and person-centred organization of care, as well as cultures and customs that oppose the presence of companions, especially men, during labour and birth [10].

Papua New Guinea (PNG) is a lower middle income country and the most populous in the Pacific Region with an estimated population over 9 million. More than 800 languages are spoken across the country [11]. The birth rate in PNG is over two times greater than Australia (27 per 1000 people compared to 12 per 1000 people) [12]. Maternal and newborn health outcomes in PNG are poor [13], and compounded by multiple factors including an underfunded health system, chronic workforce shortages, limited medical equipment and supplies and high aid dependency [14]. In addition, the majority of the population live in rural areas, which means that access to care can be difficult, particularly for villages situated within mountainous terrain and across scattered islands. Across PNG, there are only 0.6 physicians, nurses and midwives per 1000 population [15], well below the WHO recommendation of 4.5 adequately trained health providers per 1000 for universal health coverage [16]. There are very low numbers of critical maternity provider cadres, such as midwives [17]. Skilled health providers are often concentrated in urban and peri-urban areas, with much lower numbers in rural and remote areas. Promoting and facilitating the presence of companions may support this workforce gap and provide women with greater support during labour and birth, particularly in an under-resourced and overburden health system.

The national guideline for Obstetrics and Gynaecology in Papua New Guinea [18] specifically mentions that women should have continuous companionship throughout labour, although it does not specify who the companion may be. In PNG, the term ‘wasman/wasmeri’ (guardian) is often used to refer to a partner, family member or friend who provides support and care to a person in a health facility. Wasman/Wasmeri are rarely allowed in a labour ward or with a women during labour or birth. Most health facilities in PNG do not provide meals for patients and wasman/wasmeri will bring in food and other items needed, such as, bedsheets and medicines. A study conducted in 2011 at the Port Moresby General Hospital (the national tertiary referral hospital in PNG) found that whilst women wanted support people present, there were multiple barriers to enabling this to be a reality including an unsupportive organisational culture and reluctance from maternity staff [19].

In determining whether companionship during labour and birth is a feasible intervention to improve the quality of maternal and newborn care in PNG, it is important to understand the perspectives of women and those providing maternity care. The aim of this study was to describe perspectives and experiences of women, their partners and health providers regarding labour companionship, to identify enablers and barriers and develop a framework for implementing this intervention in PNG health facilities.

## Methods

### Ethics statement

The study received ethical approval in PNG from the PNG Institute of Medical Research’s Institutional Review Board and the National Department of Health Medical Research Advisory Committee (19.16), and in Australia by the Alfred Hospital Human Research Ethics Committee (267/19). All participants provided verbal and written consent to participate in the study.

### Study design and setting

A mixed methods quality improvement project was undertaken in five health facilities based in East New Britain, PNG. We used three methods for data collection: 1) facility audits, 2) labour observations and 3) interviews with women, their partners and maternity providers. East New Britain is a rural province located in the New Guinea Islands region, north of the mainland, with a population of approximately 400,000 [20], and an estimated 10,000 annual births [21]. Approximately, 60% of births occur in health facilities, above the PNG national average of 36% [22]. The province is similar to much of PNG in terms of disease profile and geography, including high rates of unintended pregnancy, low rates of contraception use [23], high burden of reproductive infections [24], and gaps in childhood immunization coverage [25]. East New Britain’s mountainous interior renders some communities accessible only by walking tracks, and coastal villages generally only accessible by water. In contrast with most of PNG, there is relatively good road access for the most densely populated districts.

This study was co-designed with the East New Britain Provincial Health Authority, local health services, clinicians and community members, using a Partnership-Defined Quality approach [26]. We report these findings according to the consolidated criteria for reporting qualitative research (COREQ) checklist (S1 File).

### Participants

The Provincial Health Authority nominated five facilities to participate in the study as they collectively represent at least 70% of the maternity services in the province, and include tertiary health services through to small remote facilities, as well as a mix of government and faith-based managed services (Church Health Services provide around 50% of health services across PNG). Women were eligible for labour observation if they were over 16 years of age, admitted for childbirth, and willing to participate and provide informed consent. Women were not eligible if they were presenting for reasons other than childbirth, immediately transferred to another facility or taken straight to theatre, or unable to provide consent. Recent studies have reported typical demographic and social characteristics of women attending these clinics, including very few having completed education beyond primary school and many were not in paid employment [23].

For the qualitative interviews, we purposively recruited women, their partners and maternity care providers. Women and their partners were recruited from facility postnatal wards, the day after birth. Researchers approached women and their partners, provided information about the study face-to-face with potential participants and asked them if they were interested in participating. Women were eligible if they were over 16 years of age and had recently given birth in one of these five facilities. Partners of these women, over 16 years of age, were eligible to participate. Maternity care providers in each of these facilities were also informed about the study via face-to-face discussions and invited to participate. All participants that were approached agreed to take part and no participants dropped out. Women and maternity providers who were observed during labour observations were not the same as those interviewed.

### Data collection

A mixed-methods approach to data collection was used to understand the ‘how’ and ‘why’ factors related to the phenomena of labour and birth companionship [27]. In this paper, we use the term companionship to refer to any support provided by a support person during labour and/or birth. Individual semi-structured interviews were used to ascertain rich descriptions of perceptions and experiences surrounding companionship. Data were collected between September 2019 and February 2020. Researchers with experience in maternal and newborn clinical care and research completed a two-week qualitative research training program prior to data collection. During the training program, interview guides were refined and piloted with community members and health providers.

Interviews with women, their partners and maternity providers were undertaken individually. All participants were interviewed in a quiet, private location away from the maternity wards, in a private room at the health facility or under a tree outside. All participants provided witnessed voluntary verbal and written informed consent. Only researchers and participants were present during interviews. Interviews lasted 30 to 60 minutes and were conducted by gender concordant researchers (male and female trained Papua New Guinean researchers) (PM, PH, RS, DK) using semi-structured interview guides (S2 File). Depending on participant preference and languages spoken by researchers, interviews were conducted in English (for maternity providers only), Tok Pisin (“Pidgin”—the main language spoken throughout PNG) or Tok Ples (languages spoken in the local community). English and Tok Pisin are official national languages. Whilst thematic saturation was reached prior to the completion of data collection, we continued sampling and conducting interviews to ensure we captured women, their partners and provider perspectives from all five facilities along the care pathway, from a remote community health post to a tertiary hospital.

Women and partners were not known to researchers before the study. Some maternity providers were known to the researchers as a result of previous research undertaken in facilities. Interviews were transcribed verbatim directly into Tok Pisin and then into English to ensure accurate translation of meaning. Field notes were taken by researchers and cross-checked with participants following interviews, there was no further follow up with participants.

Facility audits (S3 File) were completed in all five facilities and explored whether the birth suite offered adequate privacy to facilitate companionship, noting the presence of individual rooms, room dividers or curtains, and whether curtains were gaping or not. Labour observations were completed with 30 women in four of the five facilities, using a validated tool [28]. Observations were not conducted at a remote community health post as no birth had taken place in the facility in the 18 months prior to data collection. Women who participated in observations were different to the women who participated in interviews. Labour observations were continuous observations of women during labour by female study researchers with clinical backgrounds (nurses and community health workers) from admission, through labour and birth until 1 hour postpartum. All women and providers caring for women in labour provided verbal and written consent. Researchers strictly observed care provided and at no point intervened in care provision, except in the event of an emergency where assistance would be called for if needed. The presence of a companion during labour and or birth was the most important measure observed for this study.

### Data analysis

#### Quantitative data analysis

Univariate analyses were conducted using variables from facility audit and labour observation data. Variables included privacy, the presence of a companion during labour or birth and whether women were offered to have a companion present. Descriptive analyses were completed using Stata data analysis software (StataSE15) [29].

#### Qualitative data analysis

**Fig 1:**
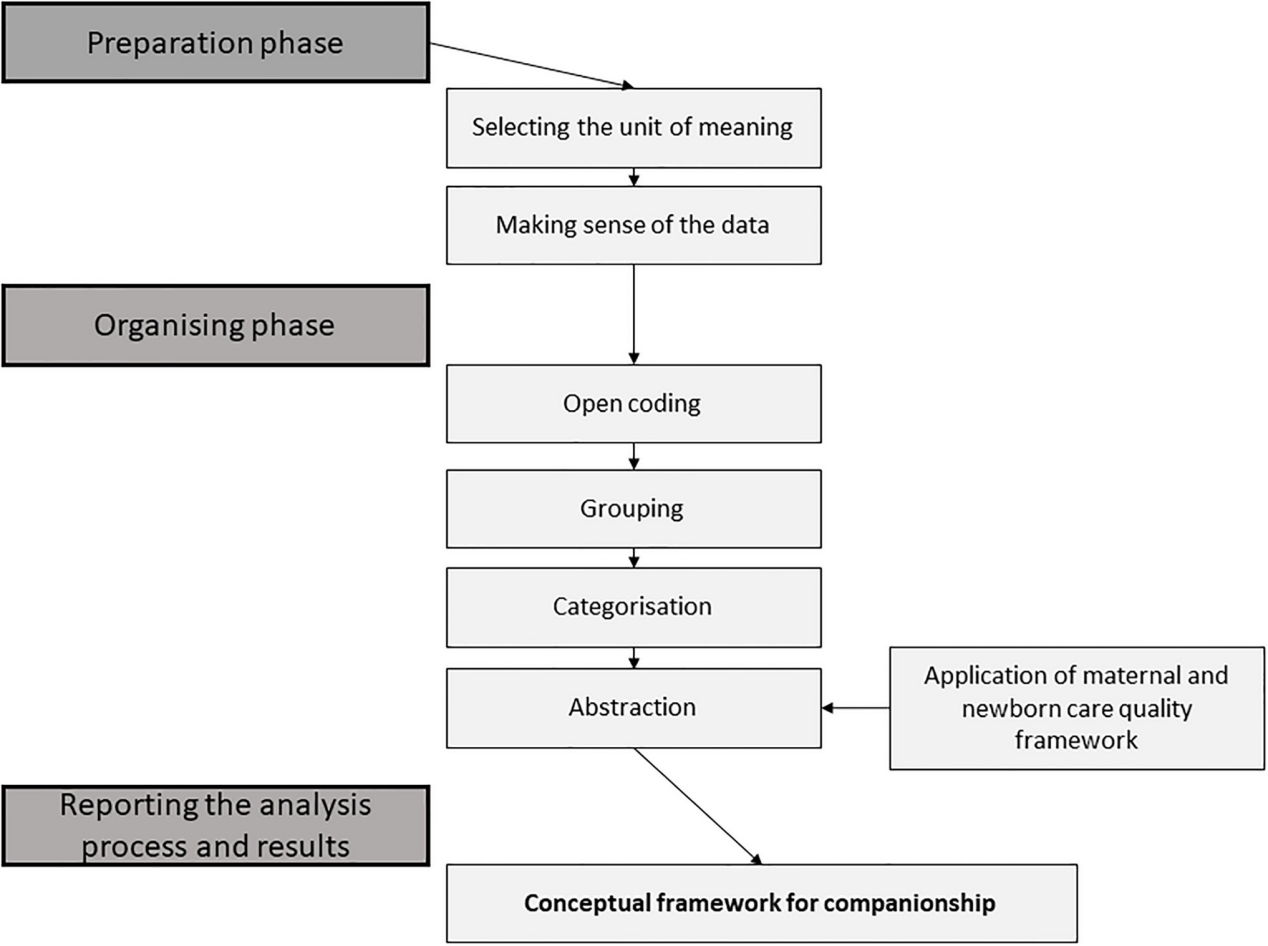
Preparation, organizing and reporting stages of content analysis. Adapted from Elo and Kyngas^31^.

Conventional content analysis was used to analyse qualitative data [30]. Content analysis was chosen as it offered a systematic and objective approach to describing a phenomena (companionship) and a process for developing a framework for companionship [30,31]. QSR NVivo software was used to manage the qualitative data [32]. The content analysis involved three phases: preparation, organising and reporting [31] (Fig 1). The first phase (Preparation) involved an inductive analysis to identify key factors associated with companionship. The inductive analysis involved reading and re-reading segments of transcripts related to companionship. Sections of text, also known as units of meaning–sentences and paragraphs–were identified.

The second stage of the analysis (Organising) involved making sense of the data by re-reading the units of meaning, open coding, creating categories and abstraction [31]. Coding was conducted jointly by the Australian and PNG research team (AW, RS, PH, PM, DK). Transcripts were open coded and a coding framework iteratively developed and discussed between researchers; when data didn’t fit into existing codes, new codes were added. Once all relevant data was coded, coded units were examined, compared and grouped into categories. Categories were then reviewed and collapsed into similar and dissimilar categories, and higher order headings applied [31]. Categorical organising of the data occurred through a process of constant comparison between categories to interpret what units belonged together versus those that did not [33]. The abstraction process enabled generation of content-characteristic titles to name categories [31]. To assist with the abstraction process, we used the conceptual quality care framework by Austin el al [34], which builds on the Donabedian framework [35]. We used this framework given its specific relevance and application to understanding the enablers and barriers to quality maternal and newborn care. The framework enabled us to arrange the categories into interconnected inputs at the community, facility and provincial levels (structures) that lead to the delivery of high quality care (processes) and result in increased companionship during labour and birth, improved care experiences and maternal and newborn health outcomes (outcomes) [34].

**Fig 2:**
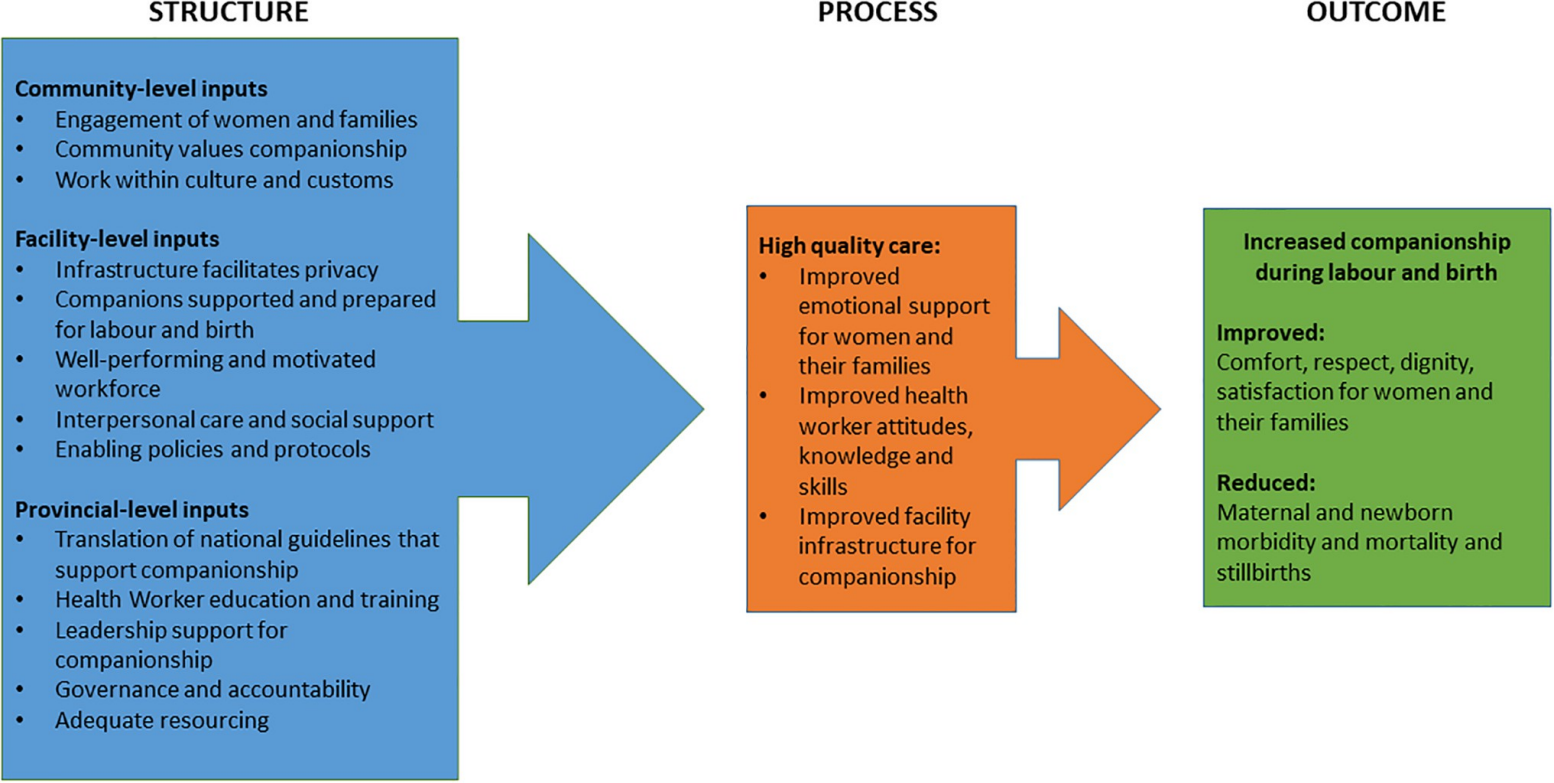
Conceptual framework for enabling companionship.

The third stage of the analysis (Reporting) involved developing a conceptual framework to outline the components needed for companionship during labour and birth in East New Britain. Drawing on the interconnected components of the Austin framework [34], we reviewed and compared the categories and sub-categories of the content analysis to develop a framework which described the structures and processes required to achieve companionship as an outcome (Fig 2).

During the data analysis process, qualitative data were also discussed at a two-day Quality Maternal and Newborn Care Workshop held in East New Britain in November 2020, attended by 35 healthcare workers, managers and community members, which provided an opportunity to member-check data collected and provide input into preliminary findings.

## Results

Five facility audits, 30 labour observations and 29 interviews with women, their partners and health providers provided three different sources of information about companionship during labour and birth. Table 1 shows sociodemographic characteristics of participants. All participants were aged over 18 years.

**Table 1:** Sociodemographic characteristics of interview participants.

	Women N = 13	Partners N = 7	Maternity care providers N = 9	Total N = 29
**Age range (years)**	21–30	24–33	32–54	21–54
**Facility** • Tertiary hospital • Secondary hospital • Rural hospital • Health centre • Community health post	5 4 2 2 0	2 2 1 2 0	2 2 2 2 1	9 8 5 6 1
**Cadre** • Midwife • Nurse • Community Health Worker	N/A	N/A	5 1 3	5 1 3

### Facility audits and labour observations

Facility audits found that most facilities offered limited privacy in the labour ward. One facility had separate cubicles with curtains, two had curtains between beds and two offered no privacy measures–although one of these facilities did have a private room for physical examinations of women. No facilities had formal policies regarding companionship, although one facility (20%) had a sign displayed at the maternity unit entry noting strictly no visitors allowed. Labour observations showed that only 30% (9/30) of women had a companion present during labour and 10% (3/30) during the birth. Only seven women (23%), were invited by the staff to have a labour companion present. All women who were offered a companion and two-thirds of those who had a companion present were in catholic run facilities. There was no association between facilities that had adequate privacy and the offer or presence of a companion.

### Interviews

Categories associated with companionship were grouped according to structural inputs [34] informed by the Austin et al. framework [34] at the community, facility and provincial level. Categories are supported by verbatim quotes indicated by italics, from numbered participants (Women (W) 1–13, Partners (P) 1–7 and Health Providers (HP) 1–9).

### Community

#### Women and their families value companionship

Women expressed a desire to have a companion present, particularly a female one, to support them during labour and birth, and also help in caring for their babies once born. As one woman described, “*They must let our wasmeris*, *our mothers or sisters to come with us to the birth room*, *to take care of our baby” (W12)*. When women were not able to have their chosen support person present some described begging for them to stay, *“They [health providers] sent my mum out but I asked for her to come in to stay with me*. *They told me that other people are not allowed to be in the labour ward*. *I begged them to let her stay with me so they allowed her to stay” (W11)*.

Some maternity providers supported women’s desire for companionship and recognised that the choice of companion was up to the women. As one health worker explained, “*It depends on individual mothers*. *If they want the partner to be with them*, *we don’t say no*, *that’s their choice*, *whoever she chooses to come” (HP8)*. Many partners expressed a desire to be present and support their partner during labour and birth, *“I wanted to come*, *I wanted to stay with her (P1)”*. Partners felt it was important for their wives to know they were there and it would make them feel more relaxed, *“My wife needs my presence to stay with her during the time of childbirth*, *so when she gives birth and is feeling pain*, *she will know that I am beside her*, *she will feel at home and release and give birth” (P2)*. Partners and health providers also expressed that it was important for partners to be present so that they understood what women go through during labour and birth.

#### Companionship provides benefits

Women described how companions could support them with bathing, breastfeeding and other tasks. As one woman explained, *“My mother or sister can help clean my baby and give [me the] baby for breastfeeding*, *whilst the nurse is removing the placenta” (W12)*. Many health facilities do not routinely provide food and so companions play an important role in providing food, as this health provider noted, *“We call out for the wasmeris to bring their meal” (HP9)*.

Health providers noted that companions could support mothers in a variety of ways, such as, offering words of encouragement and assisting with pain relief, *“They (companions) should talk nicely and support her*, *help her by rubbing her back or offering fluid or sponging her” (HP9)*. Partners also noted that they could provide physical support if present, *“I would have rubbed her back because she had requested me to do that before she went in for labour*. *She had said ‘rub my back which will cool down the pain’” (P4)*.

Partners and health providers noted that companions could assist with communication, including translation for women who did not speak Tok Pisin. As this partner explained, *“I could talk to the health care worker*. *I could ask some questions*, *understand what is going on” (P4)*. This health worker explained how companions could assist with language barriers, *“Especially the ones who are not educated or coming from the more remote areas*. *They don’t understand Pidgin so we have to get somebody to explain*. *If we don’t have anybody*, *we get their wasman/wasmeri in to explain to them” (HP9)*. Partners also described how they could advocate on behalf of women for attention and pain relief, even if this was not supported by health providers, *“When she was in pain*, *I went and asked the nurses for some medicine to ease the pain but the nurses said ‘this is labour pain and we would only be wasting medicines’” (P4)*.

Health providers believed that companionship improved labour progress for women. They noted that when partners were present, this made women feel at ease and they gave birth more quickly, *“When the man is not present with the mother during labour and childbirth*, *this affects the mother so she does not give birth quickly but when she sees her man present*, *she gives birth quickly” (HP2)*. Another health worker also noted improved labour progress, *“I see that it helps the mother in giving birth quicker*. *This is because some men*, *when they are there*, *they support their wives*, *they help them by talking to them” (HP5)*.

#### Companionship is an opportunity for partners to be aware of labour and birth processes

Health providers spoke about how the presence of partners could lead to greater understanding of what happens during labour and birth. Not only greater recognition of the pain that women go through but also an opportunity for partners to witness the birth of their child, *“I have a couple of men that really enjoyed it… they have never had such an experience witnessing their wife’s birth*…*You can really see their facial expression that they are feeling sorry for the mother and at the same time*, *watching the birth you can see the enjoyment on their face” (HP6)*.

#### Impact of cultures and customs

There were a small number of partners who explained that cultural customs and taboos prevented them from being present during labour and birth. As one man explained, *“I am not with her because of customs”* (P7). Health providers also noted that some cultural groups may preclude some men from attending, *“I am not sure if it is against the Tolai community’s traditional customs*, *but for us in Pomio [a different cultural group]*, *men are totally not allowed to be near women labouring or giving birth” (HP2)*. However, some providers noted that they were seeing the effects of health programs on encouraging companionship, *“I am seeing that some health education programs are encouraging men to be involved during labour and childbirth” (HP2)*.

### Facility

#### Health provider support for companionship

Some health providers expressed their own personal support for companions, *“I think we should allow men to come and support their wives during birth” (HP3)*. Other health providers supported companionship but not for all. One health worker explained how they only supported educated partners to be present, *“We only allow educated husbands*, *they can understand mothers and the process of childbirth” (HP4)*. Health providers expressed that support for companionship needed to begin at antenatal care, “*Yes we encourage support people*, *I think it starts from ANC (antenatal care)…the support person should be there from the start of ANC*” (HP7).

#### Health facilities need to prepare and support companions

While some health providers saw value in companionship, many commented that there needed to be some prior preparation to ready companions for the labour and birth process. Health providers described examples of where companions would not know how to help, may mistreat women and may not cope with seeing what occurred during labour and birth, *“We encourage the mothers to bring their husbands to come and we teach them what to expect in the labour room*, *we prepare the birthing partner” (HP8)*. Another provider explained how companions can mistreat women during labour, *“I’ve sent mothers out*, *literally a mother slapped her own daughter and I said “stop it*, *go outside*. *You are not going to stay here*.*” (HP4)*.

#### A lack of privacy limits companionship

A lack of adequate privacy was a significant barrier to companionship. Birth suites would often have multiple women in labour, giving birth in the one room, with little privacy between beds. Some facilities had curtains but these would often be open or gaping. As this health worker explained, *“If there are two mothers giving at the same time*, *we don’t allow them [partners] because there is not proper privacy*. *Only when there is one mother in there*, *then we allow the partner to go in”* (HP5). Partners also recognised infrastructure limitations, *“I wanted to go inside with my wife but then I knew that there were other pregnant mothers in the birthing room as well so I stayed outside”* (P5).

Health providers spoke about the availability of private rooms, generally used for conducting vaginal examinations, that could also be used during labour and birth to enable male partners to be present. As one provider explained, *“We use the private rooms for PV [per vaginal] examinations…their husbands can be in there too” (HP9)*. Partners spoke about the use of private rooms to enable male partners to be present *“They should extend and build rooms so that when couples come*, *the husband can be in the same room with his wife during birth*. *In that way*, *privacy can be maintained and other women will not be embarrassed to be seen by one another”* (P5). One facility offered a private room at a cost, where women and their partners could have a private space, sometimes referred to as a VIP room, adjacent to the birthing suite, *“there’s a private room on the side so we do allow couples there*. *We’ve been having several men coming” (HP4)*.

#### Restrictive policies around companionship

A fundamental barrier to women having support people during labour and birth were facility policies and health providers that did not support it. A women explained how her support people were turned away, *“They didn’t allow them*, *my mother and wasmeri to come*, *they came inside but the nurses told them to go and wait outside” (W10)*. Partners also spoke about how the facility did not allow them to be present, *“I was supposed to go with my wife but I might break the hospital policy” (P3)*.

Some facilities specifically did not allow male partners whilst others had a blanket rule on companions in general, *“I ask a health worker there but she said*, *‘This place is not for males*, *it’s for mothers only’” (P2)*. Some health providers confirmed these experiences and described their preference to exclude support people, “*Many mothers come with their support persons in the labour ward but personally I don’t allow support persons into the labour room*, *I ask them to stay outside” (HP3)*. There were also examples where health providers assumed that partners didn’t want to be present, without asking the women or her partner about their preferences, *“I have never come across a husband who asked to be with his wife during labour and childbirth” (HP 4)*.

### Provincial

#### Health providers need education and training

Providers spoke about the impact of education and training on their own willingness to support companionship. This provider spoke about how staff had attended a training program which included a module on companionship that had resulted in a change of practice, *“I have observed that since we attended this training*, *we are now allowing [the] male partner to come inside”* (HP5). One provider explained that during their health studies that were taught that partners could be present during labour and birth but since working in the rural facilities, they had not seen this occurring *“At school I was told that men can also attend births but in the rural outstations I only encountered mothers coming alone”* (HP2).

#### Negative attitudes to companionship need to be addressed

Only health workers noted any disadvantages to companionship, describing their concerns about companionship, including an increased risk of infection transmission to newborns, *“We don’t allow the support person to touch the baby*. *They expose the baby to infection so I think we should minimise wasmen/wasmeris”* (HP4). Some health providers felt that the presence of support people altered the interaction between health providers and women, making women behave differently and not listen to healthcare workers, *“I see labouring mothers act silly*, *they act childish*. *I send support persons outside and the mother gives birth alone with the health worker”* (HP 3).

#### Including companions may assist the workforce

Health providers noted that companions could support short staffing and workforce issues. One health worker explained how a partner was able to help during the birth and how this improved the experience for all involved, *“She asked me*, *‘Sister*! *Can my husband come into witness by birth*?*’*, *I said*, *okay*. *She got to the labour ward and I let the husband in*. *I did the vacuum while he was there*. *He was very helpful and thankful*. *At the end he said*, *‘Sister honest your work is really a blessing’*. *I said*, *yes it is*, *thank you*.*” (HP9)* Another health worker noted that although they kept husbands out due to privacy, they would ask them to wait outside and call on them if needed, *“We keep them [companions] outside till when we need their help we just call them to come and get what help we need and then they go back” (HP6)*.

In some facilities, health providers were actively encouraging women to come with a companion to provide support to the mother. As this health worker explained, *“We are allowing it now*. *Because we have come to realise that only one nurse in there*, *can’t be running in between to rub everybody’s backs*, *prepare birth bundles and then run for whatever emergencies*. *So we are asking mothers to come with a support person” (HP9)*.

## Conceptual framework

Using the findings from the three data sources, we developed a conceptual framework to outline the factors needed to enable companionship during labour and birth in East New Britain, PNG (Fig 2). Our framework was informed by the Austin et al. framework [34], and we grouped categories according to the structural inputs required at the community, facility and provincial level to deliver high quality maternal and newborn care to ultimately improve the care experience and reduce maternal and newborn morbidity and mortality, and stillbirths. This enabled us to organise and present the findings in an actionable framework that may be used to drive the changes needed to enable companionship at the facility level. It is important to note that the structural inputs are not limited to the community, facility or provincial levels and indeed intersect, overlap and may operate at multiple levels.

## Discussion

There are very few reported studies on the experiences of companionship during labour and birth from the perspectives of women, their partners and health providers, and this is the first study to include all perspectives in PNG. Facility audit data highlighted that most facilities were not able to provide adequate privacy and space for birthing women, reflected in the small number of women observed to have a companion present during labour or birth. Benefits of companionship described by women, partners and providers included encouragement, physical support and nourishment, better communication and advocacy, improved labour progress and assistance with workforce shortages. Multiple enablers and barriers were identified including provider attitudes towards companionship, the need for education and training on working with companions, infrastructure limitations including privacy, hospital policies, and culture and customs.

Global research examining companionship has identified multiple ways in which companions can support women during labour and birth [2], as well as the potential improvements in health outcomes that may be achieved [1]. In our study, women felt that companions could assist with caring for the baby after birth or support breastfeeding. Companionship during and after birth has been shown to increase rates of early and sustained breastfeeding [36,37], which may improve nutritional status of infants and young children, bringing about reductions in infant and child mortality [38]. Previously identified factors in other populations and regions that affect the implementation of companionship in facilities include little recognition of the benefits of companionship, lack of privacy, fear of increased infection, prohibitive policies and practice, cultural inclinations and poor integration of companions [2,10], and are consistent with our findings.

Our findings also align with previous research on companionship in other LMICs which has found that women desire companions, inadequate privacy is a significant barrier, health worker support for companionship is mixed and that companions can help reduce the impact of workforce gaps [7,39]. Previous studies have consistently identified that health providers and facilities that do not permit a support person to be present during labour and birth represent a major barrier to companionship [8,40]. A mixed-methods study in Kenya identified additional barriers, including if health providers believed that the companions main role was to assist staff with non-clinical tasks rather than support women, and if health providers did not trust companions [7].

In our study, health providers reported that education and training for staff and the women’s partner was needed to understand the benefits of companionship, and prepare partners for labour and birth. Staff needed support in learning how to integrate companions into their routine care and for partners, and partners would benefit from learning how to best support women. These findings are consistent with a qualitative synthesis into perceptions and experiences of labour companionship which identified the need for training, supervision and preparation of companions for labour and birth [2]. Integrated obstetric emergency simulation training may be an avenue through which to provide training to health providers on elements of respectful care, including companionship [41]. A study in Ghana that integrated specific components of respectful maternity care into training for management of obstetric and neonatal emergencies, found women were more likely to have a labour companion at endline (32%) compared to baseline (10%) [41]. However, training of maternity providers itself is not sufficient. An Ethiopian study that provided respectful maternity care training to providers, including an emphasis on companionship, found that whilst companionship initially improved, providers felt that companionship jeopardized women’s privacy and over time companionship was not upheld [42], further emphasizing the need for a multi-pronged intervention.

Whilst there are current initiatives to promote male involvement in maternal and newborn health in Papua New Guinea [43], it is important to note that discussions around promoting and enabling companionship are not limited to or dependent on male involvement. Whilst we found that male companions were less likely to be allowed to provide support during labour and birth than female companions, we need to move beyond ‘male involvement’ as the main intervention, instead emphasizing promotion of the women’s companion of choice as the solution. However, a larger study of nearly 700 women attending antenatal clinics in East New Britain found that male partners were present at 18% of appointments, and many expressed a desire to attend, indicating a shift towards greater involvement of men during pregnancy and childbirth [23]. A global review of companionship of choice during labour and birth found the presence of male partners was considered helpful for emotional reassurance, though they were often perceived to lack the skills necessary for other aspects of support needed which female companions were able to provide [10]. In keeping with our study findings, the review found that some women appreciated the presence of a male partner to witness the pain and challenges of childbirth experience by women [10].

Whilst this research was conducted before the COVID-19 pandemic, it is important to note that we have seen major disruptions to maternity care in PNG and elsewhere as a result of the pandemic [44]. Many of these changes to service delivery aim to minimise COVID-19 transmission and have included significant restrictions on the presence of support people during antenatal appointments, labour and birth and through to postnatal care [45,46]. As such, it is likely that endeavours to support and enhance companionship during labour and birth have been negatively impacted during the pandemic. However, companionship is a critical component of quality care with a direct impact on maternal and newborn health outcomes, and as such it is essential that women have access to a companion of choice during and after the pandemic [47].

## Implications for policy and research

Companionship represents an essential element for improving the quality of maternal and newborn care in PNG. The inclusion of companionship in PNG’s national obstetric guidance documents [18] is an enabler for companionship but needs translation into facility level policies and practice. Our data suggest that companionship is a complex intervention involving multiple intersecting factors. In order to address the barriers to companionship and create an enabling environment, it is not enough to address one single factor, a multi-pronged approach is required. For example, modifying hospital infrastructure to allow better privacy for women during labour and birth is essential, but without supportive facilities and health providers that allow companionship as well as partners that want to be present, it will not be possible to achieve companionship for all women who desire it.

Enabling companionship may be an avenue through which to support efforts in PNG to increase antenatal care uptake and facility birth attendance [48]. However, further rigorous research on how companionship can be scaled up in PNG, and the subsequent impact on maternal and newborn health outcomes, is needed. Our conceptual framework has highlighted that in order to achieve an enabling environment for companionship, multiple factors need to be present and operating simultaneously including positive health worker and partner attitudes to companionship, health worker education and training, adequate hospital infrastructure, accommodating hospital policies and protocols and supportive community views on companionship.

## Strengths and limitations

The small number of labour observations conducted (N = 30) provides a limited snapshot into the number of women who were able to have a support person present at labour or birth and whether they were offered a companion, in the five participating facilities in East New Britain. A previous larger study in this population reported that male partner involvement was low [23], as we found here. It is possible that the observer presence may have altered provider behavior. We were not able to explore in greater depth whether socioeconomic, age, education, employment, marital status or physical ability impacted on experiences of companionship. Previous studies have found that women who are educated, wealthy and employed are more likely to have a companion present [7,49]. We did not specifically delineate women’s desire for companionship during labour and/or birth in the labour observations. Previous research has found that women generally desire companions during labour and after birth, but less so at the time of birth [7]. It is important to examine women’s desires for companionship during labour and birth separately [7]. Lastly, the results may not be generalisable to all women, their partners and providers in PNG. However, our study provides several important findings highlight the need for future studies in other settings across PNG.

Despite these limitations, this study makes a valuable contribution to the literature regarding companionship during labour and birth in PNG. The use of multiple data sources and engagement of three key players in maternity care (women, their partners and health providers) provides comprehensive insights into perspectives and experiences of companionship in PNG. Other notable strengths include a participatory approach whereby the study concept, design and conduct was co-developed with the provincial health authority, health care providers and community members. In addition, data analysis was collaboratively conducted by researchers in Australia and Papua New Guinea. By using content analysis, we were able to build a conceptual framework for enabling companionship. Our framework has similarities to a logic model which outlines the components required for implementation of labour companionship [2], including recognition of the need for formal policies to enable companionship in facilities, structuring labour wards to allow for adequate privacy, training providers on how to integrate companions, and the need for education of companions from antenatal care onwards about how to provide emotional, practical and advocacy support for women.

## Conclusion

Our study has found that women want companions present during labour and birth and many partners want to be present. However, mixed health provider attitudes to companionship and inadequate health facility infrastructure or restrictive policies present significant barriers to enabling companionship to occur. Health providers need education and training to understand the benefits of companionship and how to prepare and support companions, so that they in turn can best support women. Supporting women to have their companion of choice present during labour and birth is critical to improving women’s experiences of care, achieving person-centered health outcomes [50,51], and improving the quality of maternal and newborn care. In order to meet international recommendations that every woman is offered a companion of her choice during labour and birth [3], multi-layered, intersecting interventions are needed at community, facility and provincial levels.

## Supporting information

S1 File COREQ table. (DOCX)

S2 File Interview guides. (DOCX)

S3 File Facility audit. (DOCX)
