# Supplementary material for: Partnership-defined quality approach to companionship during labour and birth in East New Britain, Papua New Guinea: A mixed-methods study
Source: PLOS Glob Public Health. 2022 Feb 28;2(2):e0000102. doi: 10.1371/journal.pgph.0000102 (PMC10021905; doi:10.1371/journal.pgph.0000102)
Supplement: S2 File — (DOCX) [file pgph.0000102.s002.docx]

**Supplementary document 2.**

**Interview Guide –Women**

***Demographic details (Ol askim bilong em yet)***

1. Mother’s name: (Nem bilong mama)
2. How old you? (Yu gat hamaspela krismas?)
3. Where are you from? (Yu bilong wanem hap?) What’s your current residence? (Nau yu stap long wanem hap?)
4. Baby date of birth: (Deit bilong beibi nau yu karim)
5. What’s your highest level of education completed? (Yu pinis long wanem skul mak?)
6. What is your marital status? (Askim sapos em marit)
7. Can you tell us a story about your most recent birth? (Inap yu stori long mi long nau beibi yu karim)
   1. How did you make the decision that you were ready to give birth? (Yu bin save olsem em taim bilong karim beibi olsem wanem?) What made you decide to come to this facility? (Wanem samting mekim yu laik kam long dispela hausik?)
   2. What costs were involved and how did that affect your decision? (Hamas moni/ol narapela samting, i mekim yu long kamap wantaim dispela tingting long kam long dispela hausik?)

***At the hospital (Long hausik)***

1. Tell me about what happened when you got to the hospital? (Stori long mi long ol wanem samting i bin kamap taim yu kam kamap long hausik?)
   1. How long did you wait to be seen? (Stori long mi long hamas minit/awa yu bin weit long ol wok lain kam lukim yu?) Who attended to you? (Husait i bin kam lukim yu?)
   2. Were you examined? (Ol wok lain bin sekim yu?) Was someone asked for consent for your examination? (Igat ol lain givim tok orait long ol wok lain sekim yu?) What kind of privacy did you have for your examination? (Stori long mi long wanem kain ol samting em mekim yu pilim orait long taim ol wok lain sekim yu?)
   3. How long did you have to wait for a bed? (Yu weit hamaspela minit/awa bihain long ol wok lain givim yu bet?)
   4. Were you offered food and/or water? (Ol bin givim yu wara na kaikai?)
2. Could you tell me about the labour ward/delivery room? (Inap yu stori long haus karim/rum bilong karim beibi?
   1. What privacy did you have? (Wanem kain ol samting i stap long mekim yu ino sem/poret long taim bilong karim?)
   2. Can you tell me about the cleanliness of the room? (Inap yu stori long mi long sait bilong ol pipia na lukluk bilong rum bilong karim?)
   3. What was your access to shower and toilet like? (Stori long mi long ples bilong waswas na ples bilong toilet?)
3. Tell me about who cared for you during labour and birth? (Stori long me long husait i bin was na lukautim yu long taim yu pilim pen? Na long taim bilong karim?)
   1. What did they do for you? (Ol wok lain mekim wanem long yu?)
      1. Induction, pain relief, medical review, shower etc (Ol wok lain bin givim yu marasin long halivim yu long karim? Ol pen marasin? Ol wok lain sekim yu? Ol wok lain wasim yu?)
   2. How many times were you examined? (Hamaspela taim ol wok lain sekim yu?) By whom? (Husait sekim yu?) Did you understand why you were being examined? (Yu klia long wanem as na ol wok lain bin sekim yu?) Were you asked each time? (Long wanwan taim ol wok lain sekim yu, ol bin askim?) Was someone asked for consent for your examination? (I bin gat ol lain givim tok orait long ol wok lain sekim yu?) What kind of privacy did you have for your examination? (Wanem kain ol samting i stap long mekim yu ino sem/poret long taim ol wok lain sekim yu?)
   3. What type of physical and emotional support or encouragement did the staff provide? (Wanem kain ol sapot na toktok ol wok lain i bin givim yu long mekim yu pilim gut?) What was said? (Ol wok lain i bin tok wanem long yu?) What was done?) Ol wok lain i bin mekim wanem long yu?)
   4. What did the healthcare worker say to you about what was happening during labour/childbirth? (Ol wok lain i bin tok wanem long yu taim yu wok long pilim pen? Na taim yu karim?) Were updates provided? (Ol wok lain i bin wok long toksave long yu long wanem samting i wok long kamap long yu long dispela taim?) Were you able to ask questions? (Yu bin pilim isi long askim ol askim long dispela taim tu?)
   5. Who was there at the birth? (Husait i bin stap long taim yu karim?) How many? (Hamaspela lain i bin stap?) (healthcare workers, students, family, support people) (Ol wok lain bilong hausik, sumatin bilong hausik, ol femili, ol sampela lain bilong halivim) Were you able to choose your own support person? (I bin isi long yu makim wanpela long kam stap wantaim yu long taim bilong karim?) Who did you choose and why? (Yu bin makim husait? Bilong wanem yu makim em?)
   6. Were you able to get into a comfortable position of your choice during labour and birth? (Yu bin pilim isi long makim wei yu laik stap long laik bilong yu yet, taim yu pilim pen? (Na long taim bilong karim?)
4. Can you tell me about the delivery of the baby? (Stori long mi long taim yu karim beibi?)
   1. Any emergencies, instruments? i.e. forceps/vacuum, blood transfusions, caesarean (I bin gat sampela bikpela hevi/birua kamap long taim bilong karim? Ol wok lain i bin usim sampela ol samting long halivim yu long karim? Kain olsem ol usim ol samting olsem spun/kap halivim beibi kam aut, givim bulut, katim bel bilong mama na kisim)

***After the birth (Bihain long karim)***

1. Can you tell me about what happened after your baby was born? (Inap yu stori long mi long wanem samting i bin kamap bihain long taim yu karim beibi?)
   1. What was done to you? (Ol mekim wanem samting long yu?)
      1. i.e. skin to skin contact, resuscitation, mother-baby separation, breastfeeding, perineal tear repair, mother cleaned, vital signs i.e. blood pressure etc, blood transfusion (ol putim beibi antap long bel bilong yu, ol givim win long pikinini, ol rausim beibi go long we long yu, givim susu, samapim rot bilong karim, klinim yu, sekim gut yu na beibi, givim bulut) (if mother and baby were separated, why?) (sapos ol rausim beibi long yu, bilong wanem ol mekim olsem?)
   2. What was done to the baby? (Stori long wanem samting kamap long beibi?
      1. i.e. skin to skin contact, resuscitation, mother-baby separation, breastfeeding, immunisations or other medicines for the baby, baby bathed, cord care, eye ointment (ol putim beibi antap long bel bilong yu, ol givim win long beibi, ol rausim beibi go long we long yu, givim susu, ol givim marasin sut long beibi, wasim beibi, lukaut bilong beli baten, putim marasin long ai) (if mother and baby were separated, why?) (sapos ol rausim beibi long yu, bilong wanem ol mekim olsem?)
2. Were there times when you were left alone immediately after delivery when you needed help? (Bihain stret long yu karim, i bin gat taim ol wok lain ino bin stap wantaim yu long taim yu bin laikim halivim?) Tell me what happened? (Stori long wanem samting i bin kamap long dispela taim?)
3. Immediately after the birth, what kind of support/advice were you given about breastfeeding/caring for your yourself and your baby etc? (Bihain stret long yu karim, wanem kain lukaut/gutpela toktok ol wok lain i bin givim yu long sait bilong givim susu/lukaut bilong yu na beibi?)
4. How was this birth the same or different to your other births? (Dispela karim i wankain/ino wankain long ol arapela karim bilong yu?)

***Perspectives on experience (Ol lukluk bilong yu long wanem yu bin kamap long dispela taim)***

1. If you were to describe your overall experience of labour and childbirth in a few words/a metaphor – how would you describe it? (Sapos yu bai stori long olgeta samting i bin kamap long taim yu pilim pen igo inap long taim yu karim, long wanpela o tupela tok, bai yu tok wanem?)
2. Would you recommend to your family and friends to have their baby at this facility? (Bai yu tokim ol femili na ol poro bilong yu long kam karim beibi long dispela hausik?) Why/why not? (Bilong wanem bai yu tokim ol long kam long dispela hausik? Bilong wanem bai yu no inap tokim ol long kam long dispela hausik?)

***Values around Quality of Care (Wanem yu ting em gutpela laukaut)***

1. What things do you think should happen for all mothers and babies should have during labour and childbirth? (Wanem ol samting yu ting i mas kamap long olgeta mama na beibi long taim ol pilim pen na taim bilong karim?) What things should be in place? (Wanem ol samting i mas stap?) What should the experience be like? (Long tingting bilong yu, ol mama i mas pilim olsem wanem taim ol kam long hausik long karim?)
2. What things do you think the hospital needs to work on to improve the care for mothers and babies? (Yu ting wanem ol samting hausik i mas wok moa long kamapim gutpela lukaut bilong ol mama na beibi?)
3. If you had the opportunity to speak to the health facilities about the things that make up good quality care – what are the top five things you would tell them about the quality of your care? (Sapos yu bin gat sans long toktok wantaim ol hausik long wanem ol samting i kamapim gutpela lukaut, wanem ol faivpela nambawan samting yu bai inap long tokim?)
4. What are some other things that you might like to say to the hospital about the quality of your care? (Wanem sampela ol arapela samting yu bai laik long tokim ol long sait bilong gutpela lukaut?)

**Interview Guide – Partners**

Interview guide:

***Demographic details: (Ol askim bilong em yet)***

1. What’s your wife’s name? (Wanem nem bilong meri bilong yu?)
2. How old are you? (Yu gat hamaspela krismas?)
3. Where are you from? (Yu bilong wanem hap?) Where is your current residence? (Nau yu stap long wanem hap?)
4. Baby’s date of birth: (Deit bilong beibi nau meri karim)
5. What’s your highest level of education completed? (Yu pinis long wanem skul mak?)
6. What is your marital status? (Askim sapos em marit?)

***Before the hospital***

1. Can you tell me about how you were involved in the labour and birth of your most recent child? (Inap yu stoi long mi long wanem yu bin mekim long taim meri i bin pilim pen na long taim em karim displa beibi nau em karim?)
2. At what stage were you with your wife? i.e. took wife to hospital, arrived later. (Yu halivim meri bilong yu long wanem taim bilong taim em pilim pen na laik karim stret? Kain olsem, kisim em kam long hausik, em kam pinis na yu kam bihain)
3. How was the decision made for your wife to go to hospital? What time of the day was it? (Yu kamap wantaim dispela tingting long kisim meri bilong yu long go long hausik olsem wanem?
4. What costs were involved and how did that affect the decision for her to go to the hospital? (Hamas moni/narapela samting i mekim yu long kamap wantaim dispela tingting long kisim meri bilong yuk am long hausik?)

***At the hospital***

1. Tell me about when you got to the hospital? (Stori long mi long taim yu kam kamap long hausik.) What was happening with your wife when you got there? (Wanem ol samting i wok long kamap long meri bilong yu taim yu kam kamap?)
2. Tell me about who was looking after your wife? (Stori long mi long husait i wok long lukautim meri bilong yu?) Did she already have a support person with her? (Em bin gat was man/meri wantaim em?) Who was this? (Em husait?)
3. What were some of the things that the health workers were doing to your wife when you got there? i.e. blood pressure, medicines, examinations (Wanem ol sampela ol samting ol wok lain long hausik i wok long mekim long meri bilong yu taim yu kam kamap?) (Kain olsem; ron bilong bulut, marasin na ol arapela ol sekim)
4. To what extent do you feel that your wife knew what was happening? (Igo inap long wanem mak stret yu pilim olsem meri bilong yu save long wanem ol samting i wok long kamap long em?) To what extent did you understand what was happening to your wife? (Igo inap long wanem mak stret yu kilia long wanem ol samting iwok long kamap long meri bilong yu?) Were you able to ask questions? (Yu bin isi long askim ol askim long dispela taim tu?) Were updates provided? (Ol wok lain i bin wok long toksave long yu long wanem samting iwok long kamap long meri bilong yu long dispela taim?)
5. What was the room like that your wife gave birth in? (Stori long mi long lukluk bilong yu long rum meri bilong yu karim long em?) What kind of privacy did she have? (Wanem kain ol samting i stap long mekim meri bilong yu ino ken sem/poret long dispela taim?) Can you tell me about the cleanliness of the room? Inap yu stori long mi long sait bilong ol pipia na lukluk bilong rum taim meri bilong yu kam karim?) What was her access to shower and toilet like? (Sait bilong usim ples bilong waswas na toilet i bin olsem wanem?)
6. What type of food or drink did your wife have access to? (Wanem kain kaikai/wara meri bilong yu i bin gat long dispela taim?)
7. How were you treated by the staff? (Ol wok lain bilong hausik i lukautim meri bilong yu olsem wanem?)
8. Who was there at the birth? Were you at birth? (Yu bin stap long taim meri bilong yu karim?) (If he was there - How many people were there and who? i.e. healthcare workers, students, family, support people). (Askim sapos em bin stap; hamaspela lain bin stap na husait?) Kain olsem ol woklain bilong hausik, sumatin, ol femili na ol lain bilong givim sapot) If you weren’t at the birth, why? (Sapos yu ino bin stap long taim meri bilong tu karim, bilong wanem na yu no bin stap?)
9. What kind of advice or support were you given to help your wife during labour and birth? Wanem kain ol gutpela toktok/halivim ol wok lain i bin givim long meri bilong yu long taim em pilim pen na long taim bilong karim?)
10. Can you tell me about the delivery of the baby? (Inap yu stori long mi long taim meri bilong yu karim beibi?)
    - Any emergencies, instruments? i.e. forceps/vacuum, blood transfusions, caesarean (I bin gat sampela bikpela hevi/birua kamap long taim meri bilong yu ibin laik karim? Ol woklain i bin usim sampela ol samting long halivim meri bilong yu long karim? Kain oslem ol usim ol samting olsem spun/kap long halivim beibi kam aut, givim blut, katim bel bilong mama na kisim)

***After the birth*** ***(Bihain long karim)***

1. Can you tell me about what happened after your baby was born? (Inap yu stori long mi long wanem samting i bin kamap bihain long meri bilong yu karim beibi?)
2. What was done to the mother? (Wanem samting i bin kamap long meri bilong yu?
   - i.e. skin to skin contact, resuscitation, mother-baby separation, breastfeeding, perineal tear repair, mother cleaned, vital signs i.e. blood pressure etc, blood transfusion (ol putim beibi antap long bel bilong mama, ol givim win long beibi, ol rausim beibi go longwe long mama, mama givim susu, samapim buruk long rot bilong karim, klinim mama, sekim gut mama na beibi, givim bulut) (if mother and baby were separated, why?) (sapos ol rausim beibi long mama bilong em, yu ting bilong wanem ol mekim olsem?)
3. What was done to the baby? (Stori long mi long wanem samting i bin kamap long beibi?)
   - i.e. skin to skin contact, resuscitation, mother-baby separation, breastfeeding, immunisations or other medicines for the baby, baby bathed, cord care, eye ointment (ol putim beibi antap long bel bilong mama, ol givim win long beibi, ol rausim beibi go longwe long mama, mama givim susu, ol givim marasin sut long beibi, wasim beibi, lukaut bilong beli baten, putim marasin long ai) (if mother and baby were separated, why?) (sapos ol rausim beibi long mama, yu ting bilong wanem ol mekim olsem?)
4. Were there times when your wife was left alone immediately after delivery when she needed help? (Bihain stret long tim meri bilong yu karim, i bin gat tai mol wok lain ino bin stap wanem em long taim em laikim?) Tell me what happened? (Stori long wanem samting i bin kamap.)
5. Immediately after the birth, what kind of support/advice were you given about caring for your wife and new baby? (Bihain stret long meri bilong yu karim, wanem kain lukaut/Gutpela toktok ol wok lain i bin givim yu long sait bilong lukautim bilong meri bilong yu na beibi?)
6. If you were to describe your overall experience of labour and childbirth in a few words/a metaphor – how would you describe it? (Sapos yu bai stori long olgeta samting kamap i bin kamap long taim meri bilong yu i bin pilim pen igo inap long taim em karim long wanpela o tupela tok, bai yu tok wanem?)
7. Would you recommend to your family and friends to have their baby at this facility? (Bai yu tokim ol femili na ol poro bilong yu long kisim ol meri bilong ol long kam karim beibi bilong ol long dispela hausik?) Why?/why not? (Bilong wanem bai yu tokim ol long kam long dispela hausik? Bilong wanem bai yu ino inap long tokim ol long noken kam long dispela hausik?)

***Men’s role in pregnancy and childbirth (Ol wok mak bilong man long taim mama bel na long taim mama karim)***

1. To what extent are you happy with how you were able to support your wife during labour and childbirth? (Igo inap long wanem mak stret yu pilim hamamas long wanem ol halivim yu bin inap/isi long givim long meri bilong yu long taim em pilim pen na long taim em karim?) How would you like it to have been different? (Yu bin laikim ol dispela halivim long kamap napela kain olsem wanem?)
2. What were the barriers to you being involved? (Stori long wanem ol sampela samting ibin mekim hat long yu long stap wantaim meri bilong yu long taim bilong karim?) At the facility level? (Insait long hausik?) Community level? (Insait long ples?) Family level? (Insait long femili?)
3. What are the facilitators to you being involved? (Wanem ol samting i mekim yu i laik stap long taim meri bilong yu karim?) At the facility level? (Insait long hausik?) Community level? Insait long ples?) Family level? (Insait long femili?)
4. What were the benefits for your wife of your involvement? (Yu ting wanem em ol gutpela samting bai kamap long meri bilong yu stap long dispela kain taim bilong meri bilong yu?)
5. What things do you think should happen for all mothers and babies during labour and childbirth? (Wanem ol samting yu ting i mas kamap long olgeta mama na beibi long taim mama i pilim pen na long taim bilong karim?) What things should be in place? (Wanem ol samting i mas stap?) What should the experience be like? (Long tingting bilong yu, ol mama i mas pilim olsem wanem taim ol kam long hausik long karim?)
6. What things do you think the hospital needs to work on to improve the care for mothers and babies? (Yu ting wanem ol samting hausik i mas wok moa long kamapim gutpela lukaut bilong ol mama na beibi?)
7. If you had the opportunity to speak to the health facilities about the things that make up good quality care – what are the top five things you would tell them about the quality of your care? (Sapos yu bin gat sans long toktok wantaim ol hausik long wanem ol samting i kamapim gutpela lukaut, wanem ol faivpela nambawan samting yu bai inap long tokim?)
8. How important is the role of fathers in the quality of care for mothers and babies during labour and childbirth? (Yu ting ol halivim/sapot ol papa i givim long kamapim gutpela lukaut bilong mama na beibi long taim mama pilim pen na long taim bilong karim em impoten olsem wanem?)

**Interview Guide – Health Workers**

***Demographics***

1. What’s your name?
2. What’s your highest level of education completed?
3. What’s your role at this facility? How long have you been in this role?
4. Have you worked anywhere else? Where and how long for?

***Care during labour and childbirth***

1. Can you tell me about what happens when a woman arrives in labour? i.e. admission, examinations, paperwork
2. How do you care for women in labour? How often do you do a vaginal exam? How do you use the partograph?
3. What happens if many labouring mums come in at the same time? How do you manage this?
4. Tell me about how you provide support and encouragement to a woman in labour? Are women able to have a support person during labour and childbirth? If not, why?
5. What positions do you think are more comfortable for women in labour? How are you able to help a woman get into a comfortable position?
6. How is privacy provided to a woman in labour? i.e. curtains/screens?
7. How do you explain to a woman about what is going on in labour? Do you speak to the woman? Who else do you speak to? What information is given? Can you tell me about any challenges in communicating with women or their families?
8. Can you tell me about the consent process? What procedures do you get consent for? What considerations are there for young mothers (<18 years)?
9. What common requests or demands do women have during labour and childbirth?
10. What access do women have to food and water during labour?
11. What access do they have to a toilet/shower during labour?
12. What pain relief is offered?
13. Can you tell me about what happens immediately after a baby is born?
14. What care do you provide to the baby? i.e. positioning of the baby (skin-to-skin), drying of the baby, timing of cord clamping, initial baby checks, breastfeeding, any vaccinations, Vit K
15. What care do you provide to the mother immediately after the baby is born?

***Facility births***

1. Although we have health facilities available, women are still giving birth in the villages. What do you think are some of the possible reasons why women choose to give birth in the village?
2. Which of these reasons do you think is the main reason?
3. Can you tell me how you care for young mothers (under 18 years). What are some of the issues in caring for these mothers?
4. How do you know if a mother is satisfied with the care provided? How do you know when they are not satisfied?
5. What do you think women and families expect when they come to your facility?

***Men’s involvement in care***

1. What can you tell me about men’s involvement during labour and childbirth? How are they involved? If they are not involved, why do you think this is?
2. Does this facility support men’s involvement during labour and childbirth? How?
3. Do you think men have a role to play during labour and childbirth? Tell me about this.

***Perceptions of quality maternal and newborn care***

1. What does quality maternal and newborn care mean to you? How you do you know when you are providing good quality care? How do you know when you are not?
2. What makes it easier for you to provide quality maternal and newborn care?
3. What makes it harder for you to provide quality maternal and newborn care?
4. What things do you think your health facility is doing well in providing maternal and newborn care? What things are not being done so well and need to improve?
